# Cytokine and Chemokine-Associated Signatures Underlying Dermal Invasion and Skin Metastasis in Melanoma

**DOI:** 10.3390/ijms26199334

**Published:** 2025-09-24

**Authors:** Viktória Koroknai, István Szász, Tünde Várvölgyi, Gabriella Emri, Ádám Fodor, Margit Balázs

**Affiliations:** 1 Department of Public Health and Epidemiology, Faculty of Medicine, University of Debrecen, 4028 Debrecen, Hungary; koroknai.viktoria@med.unideb.hu (V.K.); szasz.istvan@med.unideb.hu (I.S.); 2 HUN-REN-UD Public Health Research Group, Department of Public Health and Epidemiology, Faculty of Medicine, University of Debrecen, 4028 Debrecen, Hungary; 3 Department of Dermatology, Faculty of Medicine, University of Debrecen, 4032 Debrecen, Hungary; varvolgyi.tunde@med.unideb.hu (T.V.); gemri@med.unideb.hu (G.E.)

**Keywords:** melanoma, metastasis, dermal endothelium, cytokines, chemokines, Midkine, *IL1RAPL2*, *TNFRSF10A*, *IL6ST*

## Abstract

Metastatic spread remains the primary cause of mortality in melanoma. Our aim was to investigate the role of dermal endothelial cells in modulating melanoma cell invasiveness and cytokine/chemokine pattern. Primary melanoma cell lines were co-cultured with human dermal endothelial cells and assessed using Matrigel invasion assays. Invasive and non-invasive subpopulations were separated for gene expression analyses, and candidate molecules were further evaluated in patient tissue and plasma samples. Co-culture of melanoma and dermal endothelial cells revealed altered expression of several cytokine receptor genes (*CCR5*, *CXCR7*, *IL1RAPL2*, *IL4R*, *IL6ST*, *IL18R1*, *IL22RA2*, *TNFRSF10A*, *TNFRSF11B*, and *TNFRSF21*). Analysis of clinical melanoma samples showed significant downregulation of *IL1RAPL2* and *TNFRSF10A* in cutaneous metastases, whereas *IL6ST* expression correlated with Breslow thickness of the primary tumor rather than metastatic site. Proteome profiling of dermal endothelial cells revealed alterations in Midkine, GROα, MIP-3α, IL-8, and SDF-1 following co-culture with melanoma cells. Plasma measurements in melanoma patients confirmed elevated Midkine levels in skin metastases and decreased MIP-3α in metastatic disease. These results highlight potential cytokine and chemokine-mediated pathways involved in melanoma dermal invasion and cutaneous metastasis. While some findings did not reach statistical significance, concordant trends between in vitro and patient-derived data suggest their relevance and warrant further investigation in larger cohorts.

## 1. Introduction

Melanoma is an invasive, highly lethal neoplasm of melanocytes that accounts for almost 90% of skin cancer deaths worldwide [1,2,3,4,5], primarily due to its aggressive met-astatic potential [6,7]. These metastases may present as the first clinical sign of advanced disease recurrence, with reported incidence rates ranging from 2% to 20% [5,8,9]. Clinically, cutaneous metastases can resemble benign and malignant skin lesions and often exhibit atypical or unfamiliar appearances, leading to diagnostic challenges [10]. Among patients with cutaneous or subcutaneous metastases (M1a), the one-year survival rate is 59% [5].

Metastasis is a complex, multistep process involving the following: (1) the escape of tumors cells from the primary lesion; (2) invasion into blood or lymphatic vessels; (3) adhesion of circulating metastatic cells to endothelial cells of distant organs; (4) survival in a new tissue microenvironment; and (5) proliferation to establish secondary tumors [11,12]. Organ-specific patterns of metastasis are influenced by anatomic factors such as blood flow, as well as intrinsic cellular properties, including receptor expression and altered cytoskeletal dynamics [13]. In addition, tumor-associated chemokines and cytokines can activate signaling pathways that facilitate organ-specific metastasis [14,15].

Cytokines and chemokines—a subset of chemotactic cytokines—are soluble extracellular proteins that play a central role in intercellular communication. By binding to specific receptors, they regulate a wide range of biological processes, including cell growth, cellular differentiation, hematopoiesis, lymphocyte trafficking, inflammation, and immune responses [16]. Endothelial cells lining blood vessels across different organs express distinct profiles of cytokines, chemokines, and adhesion molecules, which contribute to the homing and adhesion of circulating tumor cells via receptor–ligand interactions on the tumor cell surface [17,18,19]. Endothelial cells exhibit organ-specific molecular and functional characteristics depending on their tissue of origin [20,21]. In the skin, the body’s largest and outermost organ, blood vessels play a key role in immune surveillance and response to environmental stimuli [20]. Human dermal microvascular endothelial cells (HDMECs), in particular, display unique features in metabolism, cytokine signaling, chemotaxis, and cell adhesion. For example, they express high levels of CXCL14 and CCL14, which are involved in monocyte recruitment and activation [20]. Moreover, HDMEC-derived cytokines and chemokines can promote tumor cell migration, adhesion, and invasion—key steps in the metastatic cascade [22].

Current treatment strategies for melanoma—including surgery, chemotherapy, targeted therapies, and immunotherapies—have significantly improved patient outcomes; however, important limitations persist [23]. These include adverse side effects, lack of tumor specificity, and diminished efficacy due to drug resistance [24]. The continued rise in melanoma-related mortality highlights the urgent need for novel therapeutic approaches [25]. Combining cytokines with immune checkpoint inhibitors has shown promise in enhancing anti-tumor immune responses, including melanoma [26,27].

In this study, we aimed to investigate how different melanoma cell lines influence cytokine and chemokine expression in human dermal microvascular endothelial cells (HDMECs). We also examined the expression of the corresponding receptors and evaluated the invasive potential of melanoma cells. To validate the relevance of our in vitro findings, we confirmed key observations using patient-derived tumor tissues and plasma samples. By identifying cytokines and chemokines with altered expression in endothelial cells—and elucidating their associated receptor pathways—this study may provide new insight into the molecular mechanisms underlying melanoma metastasis and support the development of targeted therapeutic strategies.

## 2. Results

### 2.1. Effect of HDMEC-Conditioned Medium on Melanoma Cell Invasion

**Figure 1:**
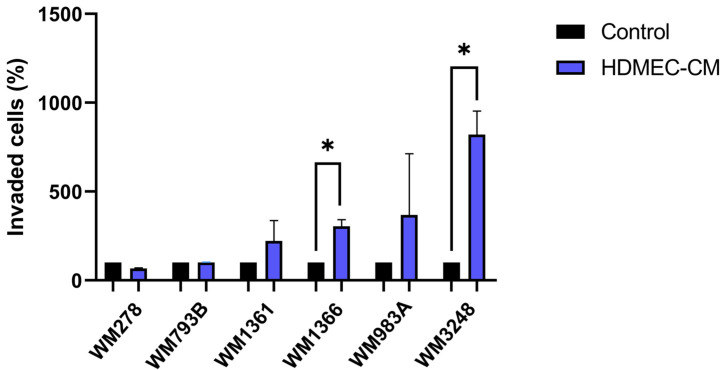
Invasive potential of six melanoma cell lines (WM278, WM793B, WM1361, WM1366, WM983A, and WM3238) in the presence of control medium or HDMEC-conditioned medium (HDMEC-CM). Asterisks indicate statistically significant differences between HDMEC-CM and control conditions (Mann–Whitney test: * *p* ≤ 0.05). Data represent the mean ± standard deviation of three independent experiments.

To investigate whether dermal endothelial cells influence melanoma cell invasiveness, six melanoma cell lines (WM793B, WM1361, WM278, WM983A, WM1366, and WM3248) were examined in Matrigel invasion chambers using HDMEC-conditioned medium (HDMEC-CM) as a chemoattractant. Two cell lines (WM278 and WM793B) showed no change in invasion compared to control conditions. Four cell lines (WM1361, WM1366, WM983A, and WM3248) exhibited a tendency toward increased invasiveness in the presence of HDMEC-CM, with statistical significance reached only in WM1361 and WM983A (Figure 1). Based on their response to HDMEC-CM, the melanoma cell lines were grouped into two categories: those with unchanged invasiveness (WM278 and WM793B) and those showing increased invasiveness (WM1361, WM1366, WM983A, and WM3248).

### 2.2. Cytokine and Chemokine Receptor Gene Expression in Melanoma Cell Lines

To assess gene expressions associated with melanoma cell invasion through the dermal endothelium, invasive and non-invasive cell populations were isolated from five melanoma cell lines following co-culture with HDMECs (WM1366, WM278, WM793B, WM983A, and WM1361; WM3248 was excluded due to cell loss). Subsequent experiments were performed with five invasive cell populations (WM1366-D^INV^, WM278-D^INV^, WM793B-D^INV^, WM983A-D^INV^, and WM1361-D^INV^; D = dermal and INV = invasive phenotype), and their five corresponding non-invasive counterparts (WM1366-D^NON-INV^, WM278-D^NON-INV^, WM793B-D^NON-INV^, WM983A-D^NON-INV^, and WM1361-D^NON-INV^; D = dermal and NON-INV = non-invasive phenotype).

Cytokine and chemokine receptor expressions were analyzed using a cytokine and chemokine receptor panel (Supplementary Table S1), and relative mRNA expression changes were then quantified as the ratio between invasive and non-invasive cell populations. Comparison of cell lines with unchanged invasiveness (WM278 and WM793B) versus those with increased invasiveness (WM1361, WM1366, WM983A) revealed trends toward higher *CCR5* and *IL22RA2* expression in the more invasive cell lines, while *IL1RAPL2, IL18R1, TNFRSF10A*, and *CXCR7* showed lower expressions. These differences did not reach statistical significance, likely due to the limited sample size, and are considered preliminary observations (Supplementary Figure S1).

### 2.3. Cytokine and Chemokine Receptor Gene Expression in Melanoma Tumor Samples

To validate our in vitro findings, we analyzed the gene expression of 10 selected candidate genes (*CCR5*, *CXCR7*, *IL1RAPL2*, *IL4R*, *IL6ST*, *IL18R1*, *IL22RA2*, *TNFRSF10A*, *TNFRSF11B*, and *TNFRSF21*) in tissue samples from three groups: (1) primary melanomas without metastasis, (2) primary melanomas with skin metastasis, and (3) metastatic skin lesions (Supplementary Table S2). Among these genes, *IL1RAPL2*, *TNFRSF10A*, and *IL6ST* exhibited apparent differences in expression between metastasis-related samples (primary melanomas with skin metastasis and skin metastases) and non-metastatic primary tumors. However, these differences did not reach statistical significance in the overall group comparison (*p* = 0.141; *p* = 0.160; *p* = 0.301; Kruskal–Wallis test) and should therefore be regarded as preliminary observations.

**Figure 2:**
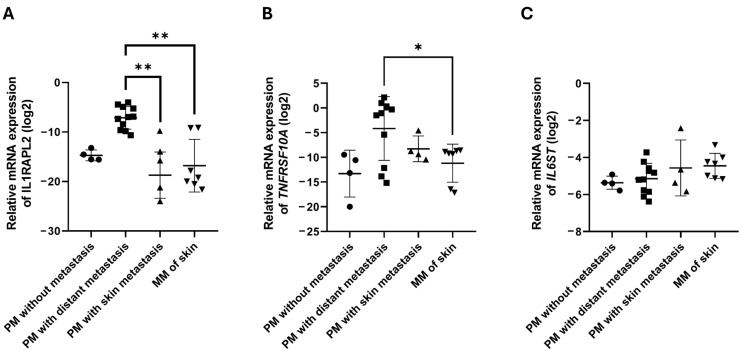
Expression of IL1RAPL2, TNFRSF10A, and IL6ST in primary melanomas and metastases. Relative mRNA expression of *IL1RAPL2* (**A**), *TNFRSF10A* (**B**), and *IL6ST* (**C**) in different melanoma tissue types: primary melanomas (PM) without metastasis (n = 4), PM with distant organ metastasis (n = 11), PM with skin metastasis (n = 4), and metastatic melanoma (MM) of the skin (n = 7). Statistical comparisons were performed using the Kruskal–Wallis test. Asterisks indicate statistically significant differences (* *p* ≤ 0.05; ** *p* ≤ 0.01).

Based on these preliminary findings, we further examined the expression of *IL1RAPL2*, *TNFRSF10A*, and *IL6ST* in primary melanomas with distant organ metastasis (Figure 2). *IL1RAPL2* expression was significantly lower in both primary melanomas with skin metastasis and in cutaneous metastases compared to primary melanomas with distant metastasis (Figure 2A). Similarly, *TNFRSF10A* expression was significantly reduced in skin metastases relative to primary melanomas with distant metastasis (Figure 2B). In contrast, *IL6ST* expression showed a moderate, non-significant increase in skin metastases compared with non-metastatic primary melanomas (Figure 2C).

**Figure 3:**
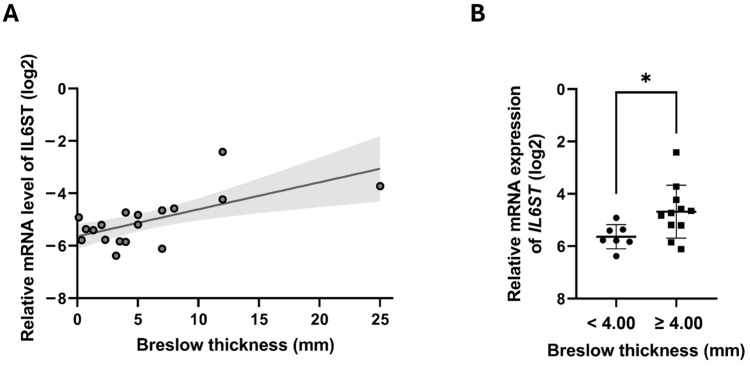
Associations between *IL6ST* gene expression levels and Breslow thickness in primary melanoma samples. (**A**) A significant positive correlation with 95% confidence intervals (gray) between relative mRNA expression of *IL6ST* and tumor thickness (in mm). (**B**) Groupwise comparison showing significantly higher *IL6ST* expression in thicker melanomas (≥4.00 mm; n = 12) compared to thinner melanomas (<4.00 mm; N = 7). Data represent the mean ± standard deviation. Asterisks indicate statistically significant differences (* *p* ≤ 0.05).

We next examined whether *IL1RAPL2*, *TNFRSF10A*, and *IL6ST* expressions correlated with Breslow thickness in primary melanoma samples (Figure 3). A significant positive correlation was observed between *IL6ST* expression and tumor thickness (Figure 3A; R = 0.582; *p* = 0.011). Consistently, groupwise comparison showed that thick melanomas (≥4.00 mm; n = 12) had significantly higher *IL6ST* expression compared to thin melanomas (<4.00 mm; n = 7) (Figure 3B; *p* = 0.027). No significant correlations were detected for *IL1RAPL2* or *TNFRSF10A*.

### 2.4. Proteome Profile of HDMECs

**Figure 4:**
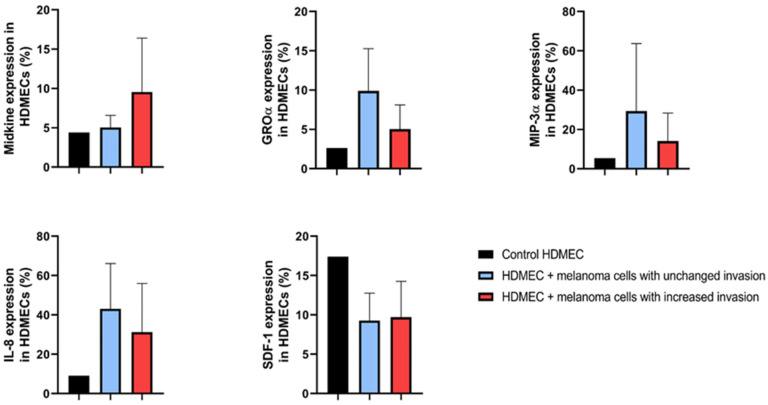
Relative protein expression profiles of human dermal microvascular endothelial cells (HDMECs) co-cultured with melanoma cell lines. Black columns represent control HDMECs; blue columns indicate HDMECs co-cultured with melanoma cell lines exhibiting unchanged invasiveness (WM278, WM793B); red columns indicate HDMECs co-cultured with melanoma cell lines exhibiting increased invasiveness (WM983A, WM1361, WM1366, and WM3248). Protein expression is shown as a percentage relative to the intensity of reference spots on the array. No statistically significant differences were observed between the HDMEC co-cultured with non-invasive versus invasive melanoma cell lines (Mann–Whitney–Wilcoxon test, *p* > 0.05).

Proteome arrays (Proteome Profiler Human Chemokine Array and Proteome Profiler Human XL Cytokine Array Kit) were used to assess the cytokine and chemokine expression in untreated HDMECs (control) and HDMECs co-cultured with six melanoma cell lines (HDMEC+WM1366, HDMEC+WM278, HDMEC+WM793B, HDMEC+WM983A, HDMEC+WM1361, and HDMEC+WM3248) (Supplementary Table S3). For this exploratory analysis, protein expression changes exceeding 10% relative to control HDMECs were considered potentially relevant. Based on this criterion, six proteins showed altered expression following co-culture, including Midkine, GROα, MIP-3α, IL-8, and SDF-1 (Figure 4).

Midkine expression tended to be higher in HDMECs co-cultured with melanoma cell lines exhibiting increased invasiveness compared to those with unchanged invasiveness; however, this difference did not reach statistical significance (Mann–Whitney test; *p* = 0.077). In contrast, GROα, MIP-3α, and IL-8 levels were lower in HDMECs co-cultured with highly invasive melanoma cell lines. Notably, SDF-1 expression was consistently reduced in HDMECs following co-culture with all melanoma cell lines relative to untreated control HDMECs.

### 2.5. Plasma Concentrations of the Candidate Proteins in Melanoma Patients

**Figure 5:**
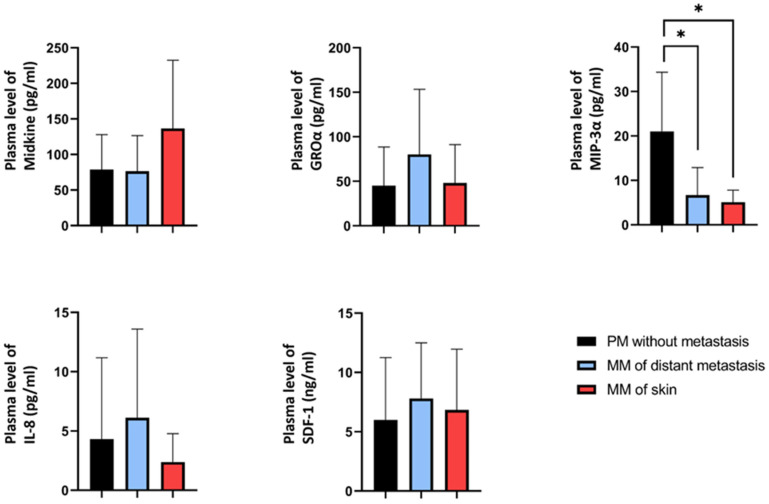
Plasma levels of selected candidate proteins in melanoma patients. Black columns represent primary melanoma without metastasis (PM, n = 10), blue columns represent metastatic melanoma with distant organ metastasis (MM, n = 20), and red columns represent melanoma with skin metastasis (n = 10). Data are presented as means of duplicates. Asterisk indicates a statistically significant difference between groups (Kruskal–Wallis test, * *p* < 0.05).

Given the moderate changes observed in the expression of Midkine, GROα, MIP-3α, IL-8, and SDF-1 proteins in dermal endothelial cells following co-culture with melanoma cell lines, we next measured the plasma concentrations of these proteins in the plasma of melanoma patients (primary melanoma without metastasis, N = 10; distant organ metastasis, n = 20; and skin metastasis, n = 10; Figure 5). Plasma Midkine levels tended to be higher in patients with skin metastasis compared to those with non-metastatic primary melanoma or distant organ metastases, although this difference was not statistically significant (*p* = 0.223, Kruskal–Wallis test). No significant differences were observed for GROα, IL-8, and SDF-1 proteins among the patient groups. In contrast, MIP-3α levels were significantly lower in both metastatic groups (distant and skin) compared to melanoma patients without metastasis (*p* = 0.039).

**Figure 6:**
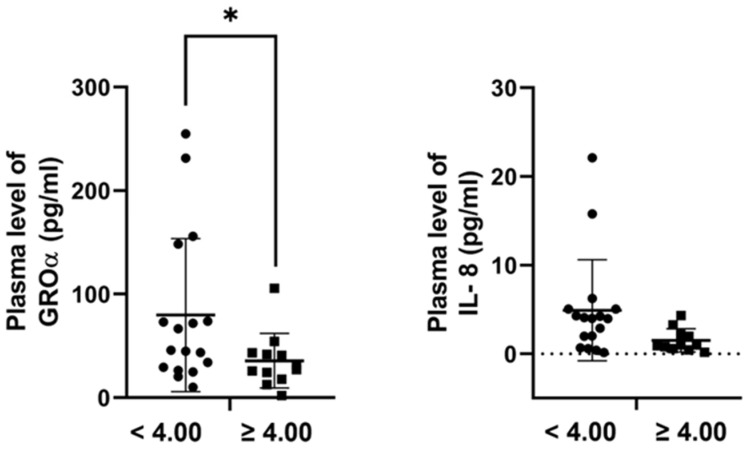
Plasma concentration of GROα and IL-8 proteins in relation to Breslow thickness of primary melanomas. Plasma concentrations of GROα and IL-8 were compared between patients with thinner (<4.00 mm; n = 18) and thicker (≥4.00 mm; n = 13) tumors. GROα levels were significantly higher in patients with thinner tumors (Mann–Whitney test, * *p* < 0.05), while the difference for IL-8 did not reach statistical significance (*p* = 0.152).

Next, we examined the association between plasma protein levels and Breslow thickness of the primary tumors. GROα and IL-8 concentrations were lower in patients with thicker melanomas (≥4.00 mm; n = 13) compared to thinner lesions (<4.00 mm; n = 18). This difference reached statistical significance for GROα (Figure 5; *p* = 0.043) but not for IL-8 (*p* = 0.152; Mann–Whitney test) (Figure 6).

## 3. Discussion

Tumor metastasis remains the leading cause of mortality in melanoma patients, as in many other malignancies. Despite significant advances in targeted therapy and immunotherapy, overall survival in metastatic melanoma remains limited [28,29]. The distribution of metastases is influenced not only by anatomical and mechanical factors, such as blood flow, but also by molecular interactions between tumor cells and the microenvironment of potential metastatic sites [22,30].

Cutaneous metastases are relatively frequent, occurring in nearly half of melanoma patients [1]. Metastatic melanoma cells often express specific chemokine and cytokine receptors that guide migration toward tissues expressing corresponding ligands [25,31]. Consequently, secondary tumor formation is shaped by both the receptor profiles of tumor cells and the ligand expression patterns of target tissues. For example, CCR10 and its ligand CCL27, produced by keratinocytes, have been implicated in cutaneous metastases formation [32,33]. Pro-inflammatory cytokines such as TNFα and IL-1β can further enhance CCR10 expression, which is associated with greater tumor thickness [32,34].

Although recent studies highlight the importance of cell-to-cell communication in melanoma development and progression, the mechanisms by which cytokines and chemokines mediate organ-specific metastasis remain incompletely understood, particularly regarding interactions with the dermal microvascular endothelium. To address this, we examined cytokine and chemokine expression in the context of skin metastasis, aiming to identify potential therapeutic targets relevant to dermal dissemination.

Using six primary melanoma cell lines, we assessed how co-culture with human dermal microvascular endothelial cells (HDMECs) influenced invasive capacity. Four of the six melanoma cell lines exhibited increased invasiveness following co-culture, while two showed no notable change. To investigate the molecular basis of these differences, each line was separated into invasive and non-invasive subpopulations, and cytokine/chemokine receptor gene expression profiles were compared. In particular, the WM278 cell line was represented by only one biological replicate, which limits the strength of statistical conclusions for this line. We nonetheless included it in the analysis to maintain group representation, but we emphasize that these findings should be interpreted cautiously. These comparisons did not yield statistically significant differences, but invasive subpopulations tended to express higher *CCR5* and *IL22RA* expression, while *IL1RAPL2*, *IL18R1*, *TNFRSF21*, *TNFRSF10A*, and *CXCR7* were generally lower. Ratios of invasive to non-invasive populations further suggested trends for *CCR5*, *TNFRSF11B*, and *IL6ST*, and a negative trend for *IL4R*, indicating potential roles in modulating melanoma invasion in the dermal microenvironment.

To assess clinical relevance, these candidate genes were examined in melanoma tissue samples. *IL1RAPL2* and *TNFRSF10A* were significantly downregulated in primary melanomas with skin metastasis and in cutaneous metastatic lesions compared to non-metastatic primary melanomas. In contrast, IL6ST expression showed a moderate but not statistically significant increase in melanomas with skin metastasis, but this increase did not appear specific to dermal invasion. In contrast, *IL1RAPL2* expression was higher in primary tumors with distant metastasis than in skin metastases, suggesting a potential role in organ-selective metastatic behavior. Although its role in melanoma has not yet been investigated, IL1RAPL2 belongs to the interleukin-1 receptor family and is implicated in neuronal development and synaptic signaling [35,36]. Its downregulation may suggest a role in modulating cell–cell adhesion and signaling, raising the possibility that loss of IL1RAPL2 contributes to enhanced melanoma cell motility or local invasion. Further studies are needed to define its function in carcinogenesis [37]. *TNFRSF10A* (also known as Death Receptor 4, DR4) is activated by TRAIL and mediates apoptosis [38]. In our study, *TNFRSF10A* expression was significantly reduced in dermal melanoma metastases compared to primary melanomas with distant metastasis. While TRIAL has shown promise as an anticancer agent, clinical outcomes have been limited, potentially due to resistance mechanisms such as the downregulation of *DR4* [38,39,40]. This could facilitate immune evasion and support metastatic progression. While we did not measure TRAIL in HDMECs, future studies should examine whether endothelial-derived TRAIL contributes to selective pressure on melanoma cells with reduced TNFRSF10A expression. IL6ST (gp130), the signal-transducing subunit of the IL-6 receptor complex, regulates apoptosis, angiogenesis, proliferation, and metastasis via STAT3 signaling [41,42]. In our study, *IL6ST* expression positively correlated with Breslow thickness, independent of metastatic site, suggesting it may reflect overall tumor aggressiveness rather than tissue-specific dissemination [43,44].

We also investigated cytokine and chemokine ligands in dermal invasion. HDMECs, Midkine, GROα, MIP-3α, IL-8, and SDF-1 showed moderate expression changes after co-culture with melanoma cell lines of higher invasiveness, but these findings did not reach statistical significance. In patient plasma, Midkine levels were higher in patients with skin metastases compared to non-metastatic or distant-metastasis cases, although the difference did not reach statistical significance. Midkine is a multifunctional growth factor that promotes epithelial-to-mesenchymal transition, angiogenesis, and NF-κB activation, and has been implicated in neolymphangiogenesis and tumor dissemination [45,46,47,48,49,50]. Interestingly, Midkine levels were lower in patients with distant metastases compared to those with skin metastases. Although this difference was not statistically significant, the elevated Midkine level potentially reflects differences in lymphatic versus hematogenous metastatic routes [51,52]. These differences may contribute to the observed patterns of Midkine expression and point to its potential role in organ-specific metastatic behavior. Plasma MIP-3α (CCL20) was significantly reduced in metastatic patients relative to those without metastasis, contrasting with prior studies suggesting a tumor-promoting role [53,54,55]. GROα (CXCL1) plasma levels were significantly higher in patients with distant metastases than in those with skin metastases, consistent with a potential role in early-stage disease and a shift toward MMP2-driven invasion in advanced tumors. GROα has a dual role in melanoma progression: it can promote tumor cell migration and invasion but also increase E-cadherin and reduce MMP2, potentially limiting metastasis despite facilitating initial dissemination [56,57,58,59]. Several important limitations must be considered. The relatively small sample size reduces the statistical power of our analyses and increases the risk of type II errors, where biologically meaningful associations may remain undetected. Although we observed concordant trends across experimental models and patient-derived data, these should be interpreted cautiously given the limited cohort size. Validation in larger, independent patient populations will be essential to confirm the robustness and generalizability of our findings.

Our findings also raise important mechanistic considerations. The downregulation of IL1RAPL2 and TNFRSF10A in dermal metastases may suggest that loss of apoptosis-related and immune-modulatory signaling supports melanoma cell survival within the dermal microenvironment. In contrast, elevated Midkine expression in skin metastases may promote epithelial-to-mesenchymal transition, angiogenesis, and neolymphangiogenesis, thereby facilitating dermal dissemination. Together, these alterations could favor a microenvironment that supports dermal colonization while enabling immune evasion.

Future studies should aim to functionally validate these hypotheses. Experimental knockdown or overexpression of IL1RAPL2 and TNFRSF10A in melanoma cell lines could clarify their role in invasion and apoptosis resistance. Similarly, in vivo models of dermal metastasis may reveal whether Midkine inhibition alters the organotropism of melanoma spread. Such studies would advance our understanding of the molecular basis of dermal dissemination and may identify novel therapeutic strategies to intercept skin-specific metastatic routes.

## 4. Materials and Methods

### 4.1. Cell Lines and Cell Culture

Melanoma cell lines derived from primary melanoma tumors (WM793B, WM1361, WM278, WM983A, WM1366, and WM3248) were obtained from the Coriell Institute for Medical Research (Camden, NJ, USA), and HDMECs (human dermal microvascular endothelial cells) were obtained from ScienCell Research Laboratories, Inc. (Carlsbad, CA, USA). Table 1 summarizes the origin and the mutation status of melanoma cell lines.

All melanoma cells were maintained in RPMI 1640 medium (Lonza Group Ltd., Basel, Switzerland) supplemented with 10% fetal bovine serum (Gibco, Carlsbad, CA, USA). The HDMECs were cultured in endothelial cell medium (ECM) supplemented with 2 μg/cm^2^ fibronectin-coated cell culture flasks (ScienCell Research Laboratories, Inc., Carlsbad, CA, USA) in a CO_2_ incubator (5% CO_2_) at 37 °C. Conditioned medium (HDMEC-CM) was collected as previously described [60]. Briefly, once the endothelial cells reached 90% confluency, the culture medium was replaced with fresh medium, and the cells were incubated for an additional 48 h. The conditioned medium was then centrifuged at 2000× *g* for 15 min and filtered through a 0.2 mm syringe filter (Merck KGaA, Darmstadt, Germany).

### 4.2. Melanoma Tissue and Plasma Samples

Fresh/frozen melanoma tissue samples were collected from the Dermatology Clinic of the University of Debrecen Clinical Centre, University of Debrecen (Debrecen, Hungary) from patients who had not received any treatment before surgical resection of the primary lesion. Blood samples were obtained from the Department of Dermatology, Faculty of Medicine, University of Debrecen, Hungary. Blood samples were transferred to the Department of Public Health and Epidemiology (University of Debrecen, Hungary) on dry ice and were stored at −80 °C until use. The study was approved by the Ethics Committee of the Hungarian Scientific Council for Health [document numbers: TUKEB 17876–2018/EKU (date of approval: 28 August 2018) and BMEU/715-1/2022/EKU (date of approval: 4 July 2022)] and was performed according to the relevant guidelines. The diagnosis of lesions was based on formalin-fixed paraffin-embedded tissue sections. The clinicopathological parameters of the melanoma tissues and plasma samples are summarized in Table 2.

### 4.3. In Vitro Invasion Assay

Melanoma cell invasion was assessed using BD Biocoat Matrigel invasion assay (pore size: 8 μm, 24 wells; BD Biosciences, Bedford, MA, USA) as previously described [61]. Briefly, melanoma cells were seeded in the upper chamber in serum-free media for the invasion assay. The lower chamber was filled with endothelial culture medium (ECM) containing 10% FBS and conditioned medium obtained from cultured HDMECs (HDMEC-CM) for the control and test experiments. After incubation at 37 °C for 24 h, the invasive cells that penetrated and attached to the bottom layer of the Matrigel membrane were fixed, stained, and counted at 200× magnification in seven different visual fields using a Zeiss Primovert inverted microscope; Carl Zeiss AG, Oberkochen, Germany.

For the co-culture of melanoma and endothelial cells, 6-well BD Biocoat Matrigel invasion chambers (pore size: 8 μm) were used as previously described [61]. A total of 2 × 10^5^ melanoma cells per well were added to the upper chamber. In parallel, HDMECs were seeded as monolayers in the lower chamber, and the cells were incubated together for 24 h. After incubation, the melanoma cells were handled separately according to their invasiveness. Invasive and non-invasive cells were removed from the bottom and surface of the membrane, respectively, with 0.5% trypsin/0.2% EDTA solution (Sigma-Aldrich Inc., St. Louis, MO, USA). WM3248 cells could not be maintained after separation for RNA isolation and were therefore excluded from further analyses. The remaining five cell lines were processed as follows: WM793B, WM1361, WM1366, and WM983A were separated in triplicate, while WM278 was separated only once due to limited cell availability.

### 4.4. Real-Time Quantitative PCR Analysis

Total RNA from each cell line was extracted with a NucleoSpin RNA kit (Macherey–Nagel, Dueren, Germany) according to the manufacturer’s protocol. The concentration and quality of RNA were determined using NanoDrop (Agilent Technologies, Palo Alto, CA, USA), and samples with a 260/280 ratio ≥1.8 were considered for further analyses. Reverse transcription of total RNA (1000 ng) was carried out using a High-Capacity cDNA Archive Kit (Applied Biosystems, Carlsbad, CA, USA) following the manufacturer’s protocol.

PCR analysis was performed using a LightCycler^®^ 480 Real-Time PCR System (Roche Diagnostics, GmbH, Mannheim, Germany). A cytokine and chemokine receptors panel based on SYBR-green (Human Cytokine and Chemokine Receptor Primer Library, RealTimePrimers.com, Elkins Park, PA, USA) was used for mRNA gene expression assays. The 2–ΔCt approach was used to evaluate changes in gene expression.

### 4.5. Proteome Profiler Assay

HDMECs were lysed in RIPA lysis and extraction buffer (Thermo Fisher Scientific Inc., Waltham, MA, USA) supplemented with protease/phosphatase inhibitor cocktail (Thermo Fisher Scientific Inc.). The protein concentration was determined using the Pierce™ Coomassie Bradford Protein Assay Kit (Thermo Fisher Scientific Inc.). The Proteome Profiler Human Chemokine Array Kit and the Proteome Profiler Human XL Cytokine Array Kit (R&D Systems, Inc., Minneapolis, MN, USA) were used to analyze protein expression according to the manufacturer’s protocol. The Azure c300 Chemiluminescent Imaging System (Dublin, CA, USA) was utilized for visualization. The data were analyzed using AzureSpot (version 2.2.167) software, and the intensity of the positive control (reference spot) was considered 100%.

### 4.6. Enzyme-Linked Immunosorbent Assay (ELISA)

The quantification of Midkine, GROα, MIP-3α, IL-8, and SDF-1 plasma levels was determined using Assay Genie ELISA kits (Assay Genie Ltd., Dublin, Ireland). The assays were carried out in accordance with the manufacturer’s protocols. The optical density of each assay was determined using an Epoch™ Microplate Spectrophotometer (BioTek Instruments, Winooski, VT, USA).

### 4.7. Statistical Analysis

IBM SPSS Statistics 29 software (IBM Corp., Armonk, NY, USA) was used for statistical analyses. The Shapiro–Wilk test was applied to assess the normality of the data. Spearman’s correlation coefficients were calculated for the correlation of the qPCR data with the invasiveness of melanoma cell lines and with the Breslow thickness of the primary melanoma samples. The Mann–Whitney–Wilcoxon and Kruskal–Wallis tests were used to compare the qPCR data, with Bonferroni correction applied for multiple comparisons. *p* ≤ 0.05 was considered statistically significant. Supplementary Table S4 shows the main steps of our analyses with the key findings.

## Figures and Tables

**Table 1:** Characteristics of primary tumour-derived melanoma cell lines.

Cell Line	Growth Phase ^†^	Subtype ^‡^	BRAF Mutation Status ^§^	NRAS Mutation Status ^¶^
WM793B	RGP/VGP	SSM	V600E	wt
WM1361	VGP	SSM	wt	Q61L
WM278	VGP	NM	V600E	wt
WM983A	VGP	n.a.	V600E	wt
WM1366	VGP	n.a.	wt	Q61L
WM3248	VGP	n.a.	V600E	wt

^†^ RGP: radial growth phase; VGP: vertical growth phase; ^‡^ SSM: superficial spreading melanoma; NM: nodular melanoma; n.a.: data not available; ^§^ V: valine; E: glutamic acid; wt: wild-type; ^¶^ Q: glutamine; and L: leucine.

**Table 2:** Clinicopathological parameters of melanoma tissue and plasma samples.

Variables	N	Variables	N
***Primary melanoma tumor samples***	**19**	***Primary melanoma plasma samples***	**10**
Gender		Gender	
Male	14	Male	6
Female	5	Female	4
Age (years)		Age (years)	
20–50	3	20–50	2
>50	16	>50	8
Breslow thickness		Breslow thickness	
<4.00	7	<4.00	8
≥4.00	11	≥4.00	2
No data	1		
Subtype ^†^		Subtype ^†^	
SSM	11	SSM	6
NM	7	NM	4
LM	1	LM	0
Metastasis formation ^‡^		Metastasis formation ^‡^	
Non-metastatic	4	Non-metastatic	10
Distant organ metastasis	11	Distant organ metastasis	0
Skin metastasis	4	Skin metastasis	0
Ulceration		Ulceration	
Present	13	Present	4
Absent	6	Absent	6
***Metastatic melanoma tumor samples***	**7**	***Metastatic melanoma plasma samples***	**30**
Gender		Gender	
Male	4	Male	18
Female	3	Female	12
Age (years)		Age (years)	
20–50	2	20–50	7
>50	5	>50	23
Localization		Localization	
Skin	7	Skin	10
Distant organ	0	Distant organ	20

^†^ SSM: superficial spreading melanoma, NM: nodular melanoma, LM: lentigo melanoma; ^‡^ Patients with follow-up periods of 36 months were included in the study.

## Data Availability

The data used to support the findings of this study are available from the corresponding author upon request.

## Supplementary Materials

The following supporting information can be downloaded at https://www.mdpi.com/article/10.3390/ijms26199334/s1.
