# Supplementary material for: Cytokine and Chemokine-Associated Signatures Underlying Dermal Invasion and Skin Metastasis in Melanoma
Source: Int J Mol Sci. 2025 Sep 24;26(19):9334. doi: 10.3390/ijms26199334 (PMC12524697; doi:10.3390/ijms26199334)
Supplement: Supplementary file 1 [file ijms-26-09334-s001.zip › Koroknai_etal_Revised_Supplementary_Table S4.pdf]

Table S4. Main steps of the analysis with the key findings.

Steps / Experiment	Description / Methods	Cell Lines / Samples	Key Results / Observations
**1. HDMEC-CM invasion assay**	Assess melanoma invasiveness in Matrigel chambers using HDMEC-conditioned medium	WM793B, WM1361, WM278, WM983A, WM1366, WM3248	WM1361, WM983A, WM1366, WM3248: increased invasion; WM278, WM793B: no change
**2. Cytokine/chemokine receptor expression in melanoma cell lines**	qPCR of invasive vs non-invasive cells after HDMEC co-culture	WM1366, WM278, WM793B, WM983A, WM1361	Trends: higher CCR5, IL22RA2; lower IL1RAPL2, IL18R1, TNFRSF10A, CXCR7 in more invasive lines
**3. Candidate gene expression in melanoma tissue**	qPCR in primary melanoma (with/without metastasis) and metastatic lesions	Primary melanomas (non-metastatic n=4, distant metastasis n=11, skin metastasis n=4), metastatic skin n=7	IL1RAPL2 & TNFRSF10A lower in skin metastases; IL6ST moderately increased
**4. Proteome profile of HDMECs**	Proteome Profiler arrays to assess cytokine/chemokine secretion	HDMECs co-cultured with 6 melanoma lines	Midkine ↑ (trend, $p=0.077$) with invasive lines; GROα, MIP-3α, IL-8 ↓; SDF-1 ↓ in all co-cultures
**5. Plasma protein levels in melanoma patients**	ELISA for candidate proteins	PM without metastasis n=10, distant metastasis n=20, skin metastasis n=10	MIP-3α significantly lower in metastatic groups ($p=0.039$); GROα lower in thicker melanomas ≥4 mm ($p=0.043$); Midkine trend ↑ in skin metastases; IL-8 & SDF-1 no significant change
