# Supplementary material for: Cytokine and Chemokine-Associated Signatures Underlying Dermal Invasion and Skin Metastasis in Melanoma
Source: Int J Mol Sci. 2025 Sep 24;26(19):9334. doi: 10.3390/ijms26199334 (PMC12524697; doi:10.3390/ijms26199334)
Supplement: Supplementary file 1 [file ijms-26-09334-s001.zip › Supplementary Table_2.pdf]

Supplementary Table S2. Relative expression levels of candidate cytokine and chemokine receptors in melanoma tumour samples.

Gene symbol	**PM[1] without metastasis (N = 4)** mean ± SD	**PM with skin metastasis (N = 4)** mean ± SD	**MM[2] of the skin (N = 7)** mean ± SD	***p* value[3]**
CCR5	0.00273 ± 0.00151	0.00433 ± 0.00421	0.01704 ± 0.02754	*1.000*
CXCR7	0.00362 ± 0.00100	0.00900 ± 0.01102	0.01853 ± 0.03054	*0.565*
IL4R	0.00361 ± 0.00158	0.00930 ± 0.01076	0.01393 ± 0.02184	*0.915*
IL6ST	0.02482 ± 0.00613	0.06715 ± 0.08034	0.05038 ± 0.02543	*0.141*
TNFRSF11B	0.00283 ± 0.00173	0.00737 ± 0.00742	0.02292 ± 0.03788	*0.747*
IL22RA2	0.00127 ± 0.00093	0.00275 ± 0.00300	0.00990 ± 0.01691	*0.786*
IL1RAPL2	0.00005 ± 0.00004	0.00002 ± 0.00003	0.00050 ± 0.00085	*0.301*
IL18R1	0.00340 ± 0.00182	0.00556 ± 0.00811	0.02987 ± 0.05135	*0.657*
TNFRSF21	0.00005 ± 0.00186	0.01478 ± 0.01333	0.03033 ± 0.03907	*0.555*
TNFRSF10A	0.00304 ± 0.00083	0.01180 ± 0.02060	0.00147 ± 0.00107	*0.160*

[1]primary melanoma; [2]metastatic melanoma; [3]Kruskal-Wallis tests were used to compare qPCR data.
