# Supplementary figures and images for: Cytokine and Chemokine-Associated Signatures Underlying Dermal Invasion and Skin Metastasis in Melanoma
Source: Int J Mol Sci. 2025 Sep 24;26(19):9334. doi: 10.3390/ijms26199334 (PMC12524697; doi:10.3390/ijms26199334)

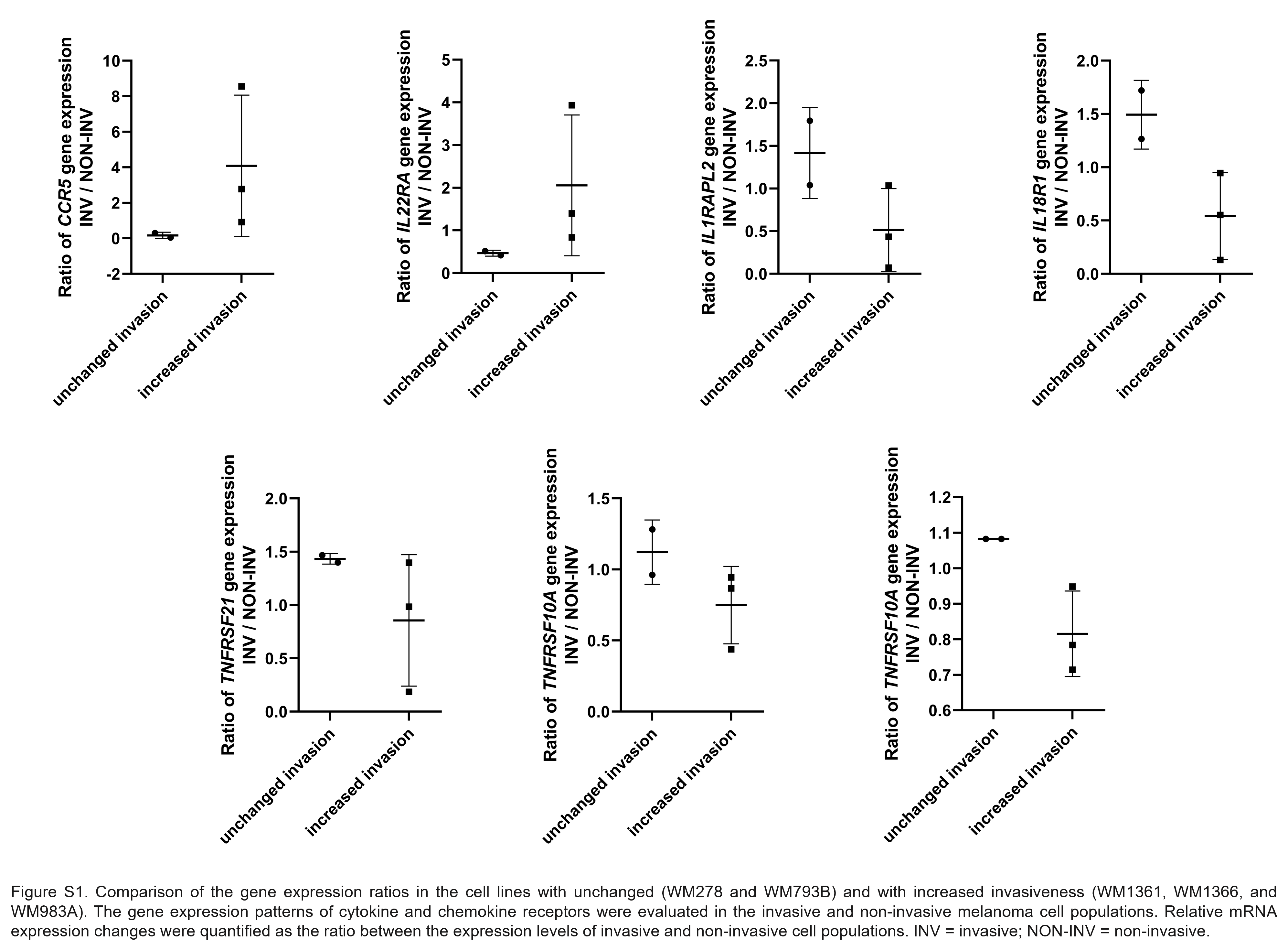

Supplement: Supplementary file 1 [file ijms-26-09334-s001.zip › Supplementary_Figure_1.png]
